# A light in the dark: Peroral cholangioscopy provides a new strategy for difficult cannulation in pancreaticobiliary maljunction

**DOI:** 10.1055/a-2096-1950

**Published:** 2023-06-12

**Authors:** Wen-Lin Zhang, Ning Zhong, Rui Ji

**Affiliations:** 1 Department of Gastroenterology, Qilu Hospital, Cheeloo College of Medicine, Shandong University, Jinan, Shandong Province, China; 2 Laboratory of Translational Gastroenterology, Qilu Hospital, Cheeloo College of Medicine, Shandong University, Jinan, Shandong Province, China; 3 Robot Engineering Laboratory for Precise Diagnosis and Therapy of GI Tumor, Qilu Hospital of Shandong University, Jinan, Shandong, China

A 56-year-old man suffered from epigastric pain for 5 days with elevated amylase (2600 IU/L), and computed tomography indicated acute pancreatitis. Magnetic resonance cholangiopancreatography showed the confluence between dilated biliary and pancreatic ducts ( Fig. 1 ), and endoscopic ultrasound indicated a 1.7-cm common channel, suggesting a pancreaticobiliary maljunction (PBM). Subsequent endoscopic retrograde cholangiopancreatography (ERCP) showed pancreatic duct opacification when conducting cholangiography ( Fig. 2 ). Considering the symptoms of acute pancreatitis and high amylase, we attempted stent implantation to conduct pancreatic juice drainage and relieve the abdominal pain.

**Fig. 1:**
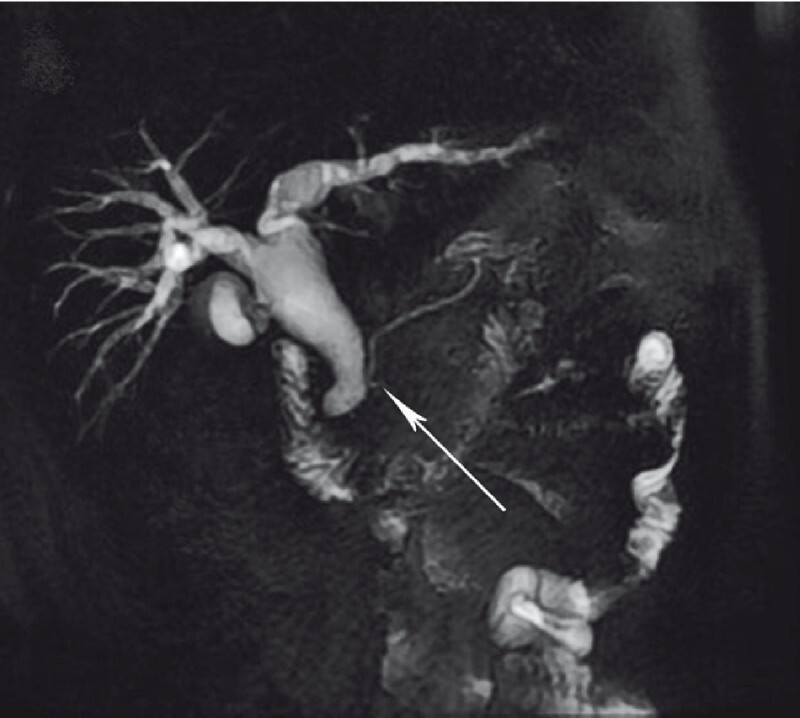
Magnetic resonance cholangiopancreatography showed the confluence between dilated biliary and pancreatic ducts.

**Fig. 2:**
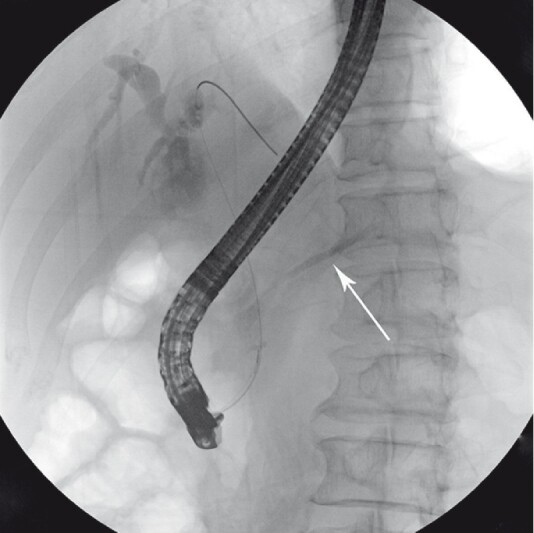
Pancreatic duct opacification when conducting cholangiography.

However, owing to the long common channel and the sharp angle, the guidewire could not be inserted into the pancreatic duct during prior attempts ( Fig. 3 a ). Therefore, a novel peroral choledochoscope (Eye-Max CDS11001, 9 Fr; Micro-Tech, Nanjing, China) with a 1.8-mm working channel was used to observe the opening of pancreatic duct under direct visualization ( Fig. 3 b , Video 1 ). The fusion junction was presented clearly inside the field ( Fig. 4 ), and the guidewire was inserted into the pancreatic duct successfully with the assistance of the peroral choledochoscope. After pancreatic duct stent implantation, the amylase gradually decreased and the patient’s recovery was uneventful during 5-month follow-up.

**Fig. 3 a:**
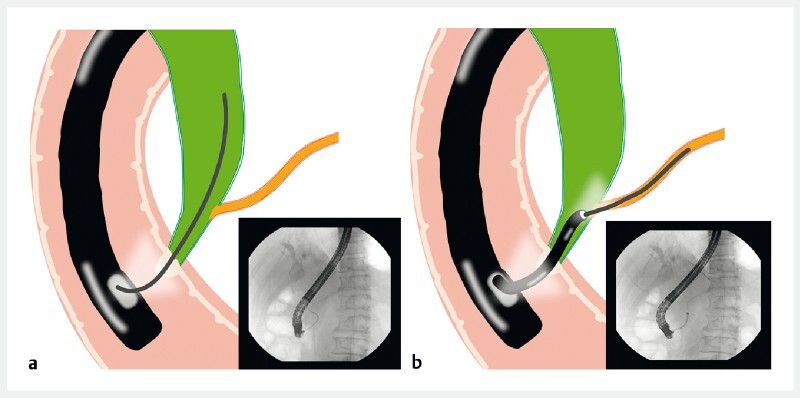
The guidewire could not be inserted into the pancreatic duct owing to the long common channel. **b** The peroral choledochoscope was used to observe the opening of pancreatic duct and insert the guidewire under direct visualization.

**Video 1** Peroral cholangioscopy-assisted pancreatic duct cannulation in a patient with a pancreaticobiliary maljunction.


**Fig. 4:**
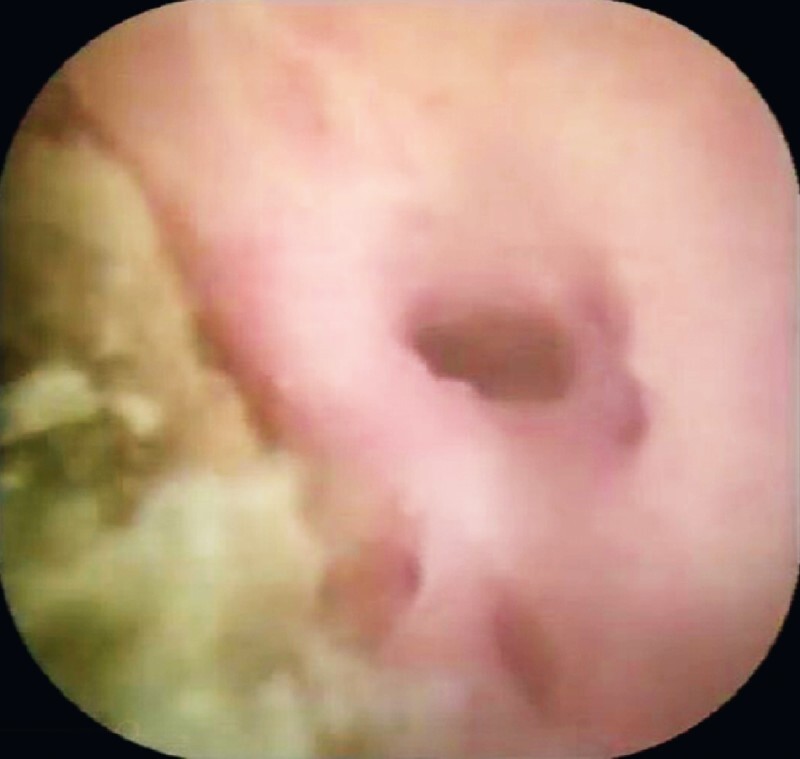
The fusion junction visible with the help of a peroral choledochoscope.

Peroral cholangioscopy has been widely applied in diagnosing pancreatobiliary diseases and shown its vital role in selective cannulation of complex biliary strictures 1 . PBM is a rare congenital malformation with the pancreatic and bile ducts united outside of the duodenal wall, resulting in dysfunction of the sphincter of Oddi and regurgitation of bile and pancreatic juice 2 3 . Endoscopic pancreatic duct stenting is an effective way to relieve the symptoms, although the diversity of anatomic variation sometimes makes cannulation difficult 4 . To our knowledge, this is the first report that applies peroral cholangioscopy in pancreatic duct cannulation in patients with PBM. Peroral cholangioscopy makes the fusion junction visible and provides a new strategy for difficult cannulation in PBM.

Endoscopy_UCTN_Code_TTT_1AR_2AI

## Notes

Endoscopy E-Videos

https://eref.thieme.de/e-videos
*E-Videos* is an open access online section of the journal *Endoscopy* , reporting on interesting cases and new techniques in gastroenterological endoscopy. All papers include a high-quality video and are published with a Creative Commons CC-BY license. Endoscopy E-Videos qualify for HINARI discounts and waivers and eligibility is automatically checked during the submission process. We grant 100% waivers to articles whose corresponding authors are based in Group A countries and 50% waivers to those who are based in Group B countries as classified by Research4Life (see: https://www.research4life.org/access/eligibility/ ). This section has its own submission website at https://mc.manuscriptcentral.com/e-videos
